# The characteristics and risk of obesity central and concomitant impaired fasting glucose: Findings from a cross-sectional study

**DOI:** 10.1371/journal.pone.0305604

**Published:** 2024-06-25

**Authors:** Iche Andriyani Liberty, Indri Seta Septadina, Mariana, Emma Novita, Resi Amalia, Esti Sri Ananingsih, Hamzah Hasyim, Laily Hanifah

**Affiliations:** 1 Department of Public Health and Community Medicine, Faculty of Medicine, Universitas Sriwijaya, Palembang, Indonesia; 2 Department of Anatomy, Faculty of Medicine, Universitas Sriwijaya, Palembang, Indonesia; 3 Faculty of Medicine, Universitas Muhammadiyah, Palembang, Indonesia; 4 Health Ministry Polytechnic, Palembang, Indonesia; 5 Faculty of Public Health, Universitas Sriwijaya, Palembang, Indonesia; 6 Universitas Pembangunan Nasional Veteran Jakarta, Jakarta, Indonesia; Faculty of Medicine, University of Belgrade, SERBIA

## Abstract

**Introduction:**

Obesity is associated with concomitant chronic conditions. An early metabolic consequence of obesity is disruption of glucose and insulin homeostasis. One of the consequences is impaired fasting glucose (IFG). Visceral fat is metabolically more harmful than subcutaneous fat, but few information is available regarding the association between the risk of abnormal glucose in increased waist circumference.

**Methods:**

This study is based on a cross sectional of 1,381 population-based from Palembang, Indonesia.

The eligibility requirements subject were to be older than 18 and consent to taking fasting glucose and lipid profile tests as well as physical exams measuring their body weight, height, blood pressure, abdominal circumference, and waist circumference.

**Results:**

The number of subjects consisting of 798 noncentral obesity with normoglycemia, 376 central obesity with normoglycemia, and 207 central obesity with concomitant IFG. The prevalence central obesity with concomitant IFG was 35.51%. In subjects with central obesity, there were significant differences in proportions based on sex, age, marital status, education, and occupation. In multivariate analysis show that the risk factors that contribute to having a significant association with central obesity with concomitant IFG are sex (female), age (>40 years), blood pressure (hypertension), and HDL-C <50 mg/dL (p<0.001). The analysis also founded that there was a significant difference in the dietary pattern of sweet foods (p = 0.018), sweet drinks (p = 0.002), soft drinks (p = 0.001) and smoking habit (p<0.001) between subjects with obesity central and concomitant IFG compared to subjects with noncentral obesity. The majority of subjects with obesity central and concomitant IFG had consuming these risky foods >6 times/week.

**Conclusion:**

The prevalence of central obesity with IFG is quite high. There are significant differences in the characteristics, lipid profile, blood pressure, dietary pattern, and smoking habit of central obesity with concomitant IFG was confirmed in this population-based observational study.

## Introduction

Obesity is an important risk factor for insulin resistance and hypertension which plays a central role in metabolic syndrome. The pathogenesis of diabetes and obesity is similar and related pathways of insulin resistance, oxidative stress (Ox-S), and pro-thrombotic and pro-inflammatory patterns [1]. The obesogenic environment stimulates overnutrition causing dysregulation of metabolic balance and ectopic fat accumulation in organs, such as the endothelium, liver, and skeletal muscle can lead to various metabolic disorders and diseases, including insulin resistance, glucose intolerance, diabetes, cardiovascular disease, and cerebrovascular disease [2–5].

Obesity is associated with concomitant chronic conditions, ranging from diabetes, dyslipidemia, to poor mental health. Its impact on the risk of stroke and cardiovascular disease, certain cancers, and osteoarthritis [6–9]. Some concomitants and risks are modifiable conditions, so lifestyle changes are needed to reduce or delay the progression of concomitants. At the same time, in many countries including Indonesia, economic growth and social changes have triggered shifts in dietary intake and physical activity at the population level, resulting in a significant increase in diabetes prevalence [10].

An early metabolic consequence of obesity is disruption of glucose and insulin homeostasis [11]. One of the consequences is impaired fasting glucose (IFG), which was introduced in the late 1990s by the American Diabetes Association (ADA) and the World Health Organization (WHO) as a stage of pre-diabetes referring to the level of fasting plasma glucose concentration above the upper normal range, but below the diagnostic limit of diabetes. The glucose range for IFG differs between organizations: the ADA glucose range is 5.6–6.9 mmol/L, while the WHO has more stringent criteria of 6.1–6.9 mmol/L. Both criteria are used and there is no consensus on IFG, especially not in children and adolescents [12, 13]. In adults, IFG is a predictor of type 2 diabetes [14, 15]. The mechanisms behind IFG are still not fully understood, but IFG results from impaired insulin secretion, which indicates beta cell dysfunction as well as increased hepatic glucose output [16]. Several studies reported the combination of general obesity and IFG was more strongly associated with diabetes risk than other studies with the same number of components [17, 18]. As visceral fat is metabolically more harmful than subcutaneous fat, but few information is available regarding the association between the risk of abnormal glucose in overweight and obese individuals and increased waist circumference [19]. Therefore, this study was conducted to identify the factors associated for obesity with concomitant IFG. Identifying the risk of IFG in obesity allows intervention with earlier precision.

## Method

### Study design and sample

This cross-sectional study involved subjects aged >18 years from Palembang, Indonesia during July-December 2022. The multistage cluster random sampling method was used to select a representative sample of the population. From a total of 17 sub-districts, Seberang Ulu and Ilir were chosen at random as the first two sub-districts. Two of the five Seberang Ulu subdistricts and two of the twelve Seberang Ilir subdistricts were randomly chosen for the second stage and 25% of the villages from each subdistrict that was chosen were randomly chosen for the third stage. All households with household members older than 18 years old were identified in the final step. The eligibility requirements: be older than 18 and consent to taking fasting glucose and lipid profile tests as well as physical exams measuring their body weight, height, blood pressure, abdominal circumference, and waist circumference. The following were the exclusion criteria for cases and controls: 1) have fasting plasma glucose >126 mg/dl; 2) currently taking oral hypoglycemic medications; 3) taking any medication that could affect how glucose, insulin, or high-density lipoprotein cholesterol are metabolized; and 4) taking any medication for obesity.

### Data collection, procedure, and measurements

The ethical guidelines established by the institutional research committee are followed in all procedures involving human beings. Through the use of questionnaires, physical exams, and blood testing, data were gathered through interviews. A standard questionnaire, information sheet, and consent form are distributed by the research team when they visit the participant’s house. If participants concur, the examination was held the following day. A blood sample was taken during a fasting period of at least eight to twelve hours. Using standardized, laboratory techniques, the levels of plasma glucose, serum total, low-density lipoprotein and high-density lipoprotein cholesterol (LDL-c and HDL-c, respectively), and triglyceride (TG) were determined. Health professionals who have previously received training carry out physical examinations, taking blood pressure and anthropometric measurements after the wearer removes their bulky clothing, belts and shoes according to examination standards. Measures of blood pressure made with a sphygmomanometer, a common piece of medical equipment. Using a questionnaire, information about sex, age, education, occupation, marital status, and dietary pattern was gathered.

### Variable definitions

Body mass index (BMI) was calculated by dividing body weight (kg) by body height (m^2^). By WHO criteria: Underweight (BMI < 18.50 kg/m^2^), normal weight (BMI = 18.50 to 24.99 kg/m^2^), overweight (BMI = 25.0 to 29.99 kg/m_2_), obese (BMI = ≥30.0). Waist circumference (WC) measurement was taken in the standing position, at the midpoint between the iliac crest and the least palpable rib precisely using non-stretchable tape. Central obesity was defined according to the WHO criteria: WC ≥ 94 cm for men and ≥ 80 cm for women.

Dietary pattern data was obtained from the quantitative food frequency questionnaire (FFQ). A photo album was used to help patients choose portion sizes. The reported intake was converted into daily consumption. Physical activity was measured using questionnaire from the International Physical Activity Questionnaire (IPAQ) [20]. The IPAQ assesses physical activity in four domains: leisure-time physical activity, household and yard activities, work-related physical activity, and transportation-related physical activity. Individuals who spent less than 600 metabolic equivalent minutes (METs) per week, which is the definition of low physical activity, were classified as inactive for this analysis. To simplify reporting, individuals who report more than 600 MET minutes per week will be referred to as “active”, whereas those with less than 600 MET minutes per week are “less active”.

### Statistical analysis

The statistical analysis was performed using the statistical program STATA version 15 (College Station, Texas 77845 USA). Categorical data represented as counts of frequencies with n (%). Multinomial logistic regression to analyze factors that were associated outcome variable (i.e., the present obesity central and IFG status) consisted of three categories: 0 = noncentral obesity normoglycemic and, 1 = central obesity with normoglycemic, 2 = central obesity with IFG. An adjusted multinomial logistic regression models were used to identify the potential factors that have a significant role in the higher risk of obesity and concomitant IFG. We checked the multicollinearity among the explanatory variables using variance inflation factor (VIF). VIF value ≤ 2.0 indicates absence of multicollinearity [21]. The data was summarized with relative risk ratios (RRR) and 95% confidence interval. Results were considered significant when p < 0.05.

### Ethical clearance

Our protocol has been reviewed and approved by the Medical and Health Research Ethics Committee (KEPKK) of the Faculty of Medicine, Universitas Sriwijaya, giving its approval to this research based on Protocol Number 073–2022 (Institutional Review Board reference). There is no financial incentive. Written informed consent was obtained from all participants.

## Result

The total number of subjects in this study was 1,381 consisting of 798 noncentral obesity with normoglycemia, 376 central obesity with normoglycemia, and 207 central obesity with concomitant IFG. Table 1 provides the characteristics of subjects with obesity and concomitant IFG. Based on the table, the prevalence central obesity with concomitant IFG was 35.51% (207 out of 583). Data analysis in this study took subjects with noncentral obesity with normoglycemia as a reference group for comparison. In subjects with central obesity, there were significant differences in proportions based on sex, age, marital status, education, and occupation. Subjects with central obesity with concomitant IFG were predominantly female (20.39%), aged > = 40 years (20%), with marital status of death divorce (32%), no school (18.29%), and not working (20.96). It is interesting that central obesity was possessed by 25.63% of subjects <40 years of age and another 9.12% with central obesity and IFG.

**Table 1:** Characteristics of subjects with obesity and concomitant IFG.

Variables	Noncentral Obesity (n = 798)	Central Obesity (n = 583)			
		Normoglycemic (n = 376)	P value	IFG (n = 207)	P value
Sex					
Male	413 (81.30)	66 (12.99)	<0.001	29 (5.71)	<0.001
Female	385 (44.10)	310 (35.51)		178 (20.39)	
Age					
<40 years	415 (65.25)	163 (25.63)	0.006	58 (9.12)	<0.001
> = 40 years	383 (51.41)	2213 (28.59)		149 (20)	
Marital Status					
Not Married	171 (85.93)	22 (11.06)	<0.001	6 (3.02)	<0.001
Married	568 (54.30)	314 (30.03)		164 (15.68)	
Divorced alive	19 (52.78)	12 (33.33)		5 (13.89)	
Death divorce	40 (40.00)	28 (28.00)		32 (32.00)	
Education					
No school	48 (58.54)	19 (23.17)	0.051	15 (18.29)	0.622
Not completed in primary school	104 (57.78)	44 (24.44)		32 (17.78)	
Graduated from elementary school	238 (58.33)	112 (27.45)		58 (14.22)	
Graduated from junior high school	169 (61.23)	68 (24.64)		39 (14.13)	
Graduated from high school	196 (57.14)	97 (28.28)		50 (14.58)	
Completed Diploma	16 (42.11)	16 (42.11)		6 (15.79)	
Completed Bachelor’s degree	27 (50.00)	20 (37.04)		7 (12.96)	
Occupation					
Not working	231 (48.43)	146 (30.61)	0.013	100 (20.96)	0.001
School	57 (79.17)	14 (19.44)		1 (1.39)	
Civil Servants	8 (34.78)	14 (60.87)		1 (4.35)	
Private employees	63 (60.00)	34 (32.38)		8 (7.62)	
Self-employed	106 (54.64)	51 (26.29)		37 (19.07)	
Farmer	203 (69.76)	55 (18.90)		33 (11.34)	
Fisherman	3 (75.00)	0 (0.00)		1 (25.00)	
Labourer/driver/helper	84 (67.20)	29 (23.20)		12 (9.60)	
Other	43 (47.78)	33 (36.67)		14 (15.56)	
Physical Activity					
Active	587 (55.85)	304 (28.92)	0.007	160 (15.22)	0.273
Less active	211 (63.94)	72 (21.82)		47 (14.24)	
BMI					
Underweight	97 (98.98)	0 (0.00)	<0.001	1 (1.02)	<0.001
Normal	605 (81.54)	94 (12.67)		43 (5.80)	
Overweight	66 (34.38)	79 (41.15)		47 (24.48)	
Obesity	30 (8.60)	203 (58.17)		116 (33.24)	
**Lipid Profile**					
Total Cholesterol					
<200 mg/dL	617 (60.91)	271 (26.75)	0.051	125 (12.34)	<0.001
≥200 mg/dL	181 (49.18)	105 (28.53)		82 (22.28)	
LDL-C					
<160 mg/dL	728 (59.62)	333 (27.27)	0.150	160 (13.10)	<0.001
≥160 mg/dL	70 (43.75)	43 (26.88)		47 (29.38)	
HDL-C					
<45 mg/dL	523 (66.88)	169 (21.61)	<0.001	90 (11.51)	<0.001
≥45 mg/dL	275 (45.91)	207 (34.56)		117 (19.53)	
Triglycerides					
<150 mg/dL	694 (60.88)	300 (26.32)	0.002	146 (12.81)	<0.001
≥150 mg/dL	104 (43.15)	76 (31.54)		61 (25.31)	
Blood Pressure					
Normotension	581 (65.28)	206 (23.15)	<0.001	103 (11.57)	<0.001
Hypertension	217 (44.20)	170 (34.62)		104 (21.18)	

There was a significant difference between subjects with noncentral obesity and central obesity with p = 0.007 based on physical activity, but not significant compared to central obese subjects with concomitant IFG. Another interesting point is that there was 1 subject with underweight BMI but had central obesity with concomitant IFG and 42 subjects (6.48%) of the sample had normal BMI but had central obesity with concomitant IFG. Although BMI obesity category dominated the central obesity subjects with concomitant IFG as many as 116 subjects (33.24%). Based on lipid profile, central obese subjects with concomitant IFG had a higher proportion of abnormal lipid profile. The proportion of central obesity subjects with concomitant IFG with abnormal lipid profile values is also quite high; respectively for total cholesterol ≥200mg/dL (22.28%), LDL-C ≥160mg/dL (29.38%), HDL-C <45 mg/dL (19.53%), and triglyceride >150 mg/dL (25.31%). Likewise with blood pressure parameters, 21.18% of central obesity with concomitant IFG had hypertension. Although partially or bivariate, physical activity, total cholesterol >200 mg/dL, LDL-C >160 mg/dL, triglyceride >150 mg/dL had a significant association with central obesity with concomitant IFG (p<0.001). In multivariate analysis (Table 2) show that the risk factors that contribute to having a significant association with central obesity with concomitant IFG are sex (female), age (>40 years), blood pressure (hypertension), and HDL-C <50 mg/dL (p<0.001).

**Table 2:** Bivariate and multivariate analysis of variables that have an association with obesity and concomitant IFG.

Variables	Central Obesity and Concomitant IFG			
	Unadjusted RRR (CI 95%)	P value	Adjusted RRR (CI 95%);	P value
Female	6.584 (4.342–9.984)	<0.001	6.372 (4.130–9.831)	<0.001
≥40 years	2.783 (1.99–3.886)	<0.001	2.852 (1.960–4.149)	<0.001
Physical Activity	0.333 (0.208–0.534)	<0.001	0.927 (0.624–1.376)	0.708
Hypertension	0.177 (0.143–0.218)	<0.001	2.228 (1.573–3.155)	<0.001
Total Cholesterol >200mg/dL	0.202 (0.167–0.245)	<0.001	1.090 (0.685–1.803)	0.737
LDL-C >160 mg/dL	0.219 (0.185–0.260)	<0.001	1.345 (0.612–2.957)	0.459
Triglyceride >150 mg/dL	0.210 (0.175–0.251)	<0.001	1.427 (0.659–3.089)	0.367
HDL-C <50 mg/dL	0.172 (0.137–0.215)	<0.001	2.751 (1.947–3.888)	<0.001

Noted: References outcome is no obesity central and normoglycemic;

Reference risk is male, <40 years, active physical activity, normal lipid profile

This study also explored the dietary pattern of the subjects (Table 3), the results of the analysis showed that there was a significant difference in the consumption frequency pattern of sweet foods (p = 0.018), sweet drinks (p = 0.002), soft drinks (p = 0.001) and smoking habit (p<0.001) between subjects with obesity central and concomitant IFG compared to subjects with noncentral obesity. The majority of subjects with obesity central and concomitant IFG had the habit of consuming these risky foods >6 times/week. Fatty food (p = 0.005), soft drink (p = 0.001), fruit consumption (p = 0.008), and smoking habit (p<0.001) were significant variables that distinguished noncentral obesity and central obesity subjects. Interestingly (Table 4), although bivariate multiple food consumption was a risk for central obesity with concomitant IFG, on multivariate only smoking habit was significant as a risk with both non-daily (p = 0.004) and daily smokers (<0.001) when compared to subjects without central obesity.

**Table 3:** Dietary patterns in subjects with obesity central and concomitant IFG.

Food Consumption/ week	Noncentral Obesity (n = 798)	Central Obesity (n = 583)			
		Normoglycemic (n = 376)	P value	IFG (n = 207)	P value
Sweet food					
< = 2	300 (58.71)	148 (28.96)	0.826	63 (12.33)	0.018
3–5	457 (57.99)	205 (26.02)		126 (15.99)	
>6	41 (50)	23 (28.05)		18 (21.95)	
Sweet drink					
< = 2	484 (60.05)	213 (26.43)	0.055	109 (13.52)	0.002
3–5	282 (57.09)	136 (27.53)		76 (15.38)	
>6	32 (39.51)	27 (33.33)		22 (27.16)	
Salty food					
< = 2	317 (58.17)	162 (29.72)	0.428	66 (12.11)	0.075
3–5	411 (57.72)	180 (25.28)		121 (16.99)	
>6	70 (56.45)	34 (27.42)		20 (16.13)	
Fatty food					
< = 2	364 (54.65)	205 (30.78)	0.005	97 (14.56)	0.877
3–5	407 (60.93)	162 (24.25)		99 (14.82)	
>6	27 (57.45)	9 (19.15)		11 (23.40)	
Grilled food					
< = 2	30 (54.55)	18 (32.73)	0.341	7 (12.73)	0.966
3–5	513 (58.97)	222 (25.52)		135 (15.52)	
>6	255 (55.92)	136 (29.82)		65 (14.25)	
Processed meat					
< = 2	29 (47.54)	24 (39.34)	0.739	8 (13.11)	0.106
3–5	378 (60.77)	162 (26.05)		82 (13.18)	
>6	391 (56.02)	190 (27.22)		117 (16.76)	
Flavored food					
< = 2	655 (57.36)	319 (27.93)	0.119	168 (14.71)	0.628
3–5	91 (58.33)	42 (26.92)		23 (24.74)	
>6	52 (62.65)	15 (18.07)		16 (19.28)	
Soft drink					
< = 2	22 (68.75)	4 (12.50)	0.001	6 (18.75)	0.001
3–5	240 (67.04)	84 (23.46)		34 (9.50)	
>6	536 (54.09)	288 (29.06)		167 (16.85)	
Instant food					
< = 2	64 (65.31)	20 (20.41)	0.098	14 (14.29)	0.425
3–5	627 (57.68)	298 (27.41)		162 (14.90)	
>6	107 (54.59)	58 (29.59)		31 (15.82)	
Vegetable					
< = 2	143 (59.83)	62 (25.94)	0.227	34 (14.23)	0.332
3–5	204 (60.71)	85 (25.30)		47 (13.99)	
>6	451 (55.96)	229 (28.41)		126 (15.63)	
Fruit					
< = 2	441 (60.16)	190 (25.92)	0.008	102 (13.92)	0.083
3–5	230 (59.13)	96 (24.68)		63 (16.20)	
>6	127 (49.03)	90 (34.75)		42 (16.22)	
Cigarettes					
Not smoking	473 (49.43)	311 (32.50)	<0.001	173 (18.08)	<0.001
Not everyday	80 (66.67)	24 (20.00)		16 (13.33)	
Everyday	245 (80.59)	41 (13.49)		18 (5.92)	

**Table 4:** Bivariate and multivariate analysis of dietary patterns that have an association with obesity and concomitant IFG.

Food Consumption / week	Central Obesity and Concomitant IFG			
	IFG		Normoglycemic	
	Unadjusted RRR (CI 95%)	P value	Adjusted RRR (CI 95%);	P value
Sweet food				
3–5	1.312 (0.938–1.836)	0.112	1.209 (0.814–1.797)	0.346
>6	2.090 (1.128–3.875)	0.019	1.765 (0.856–3.637)	0.123
Sweet drink				
3–5	1.196 (0.862–1.660)	0.283	0.844 (0.579–1.229)	0.378
>6	3.052 (1.707–5.459)	0.000	1.905 (0.991–3.665)	0.053
Salty food				
3–5	1.414 (1.012–1.974)	0.042	1.395 (0.961–2.025)	0.080
>6	1.372 (0.781–2.410)	0.271	1.176 (0.641–2.155)	0.600
Fatty food				
3–5	0.912 (0.667–1.249)	0.569	0.821 (0.582–1.160)	0.264
>6	1.528 (0.732–3.191)	0.258	1.354 (0.604–3.033)	0.460
Grilled food				
3–5	1.127 (0.484–2.623)	0.780	1.225 (0.500–3.002)	0.656
>6	1.092 (0.459–2.598)	0.842	0.925 (0.365–2.343)	0.870
Processed meat				
3–5	0.786 (0.346–1.782)	0.565	0.623 (0.261–1.484)	0.286
>6	1.084 (0.482–2.437)	0.844	0.788 (0.333–1.866)	0.589
Flavored food				
3–5	0.985 (0.605–1.604)	0.953	0.973 (0.579–1.634)	0.920
>6	1.199 (0.668–2.154)	0.542	0.958 (0.511–1.795)	0.895
Soft drink				
3–5	0.519 (0.196–1.372)	0.186	0.558 (0.198–1.568)	0.269
>6	1.142 (0.455–2.864)	0.777	1.054 (0.394–2.818)	0.916
Instant food				
3–5	1.181 (0.645–2.159)	0.589	1.032 (0.543–1.960)	0.922
>6	1.324 (0.655–2.675)	0.433	0.903 (0.419–1.903)	0.771
Vegetable				
3–5	0.969 (0.593–1.582)	0.900	0.958 (0.570–1.610)	0.873
>6	1.175 (0.769–1.793)	0.455	1.007 (0.641–1.582)	0.973
Fruit				
3–5	1.184 (0.832–1.684)	0.347	1.178 (0.808–1.718)	0.392
>6	1.429 (0.948–2.154)	0.087	1.261 (0.814–1.953)	0.299
Cigarettes				
Not everyday	2.722 (1.326–5.587)	0.006	2.908 (1.400–6.041)	0.004
Everyday	4.978 (2.991–8.284)	<0.001	4.877 (2.884–8.248)	<0.001

Noted: References outcome is no obesity central and normoglycemic;

Reference risk is ≤2 per week, and not smoking for cigarettes

## Discussion

In 35.51% of the sample (Table 1), central obesity and impaired fasting glucose (IFG) were present. This condition was more common in women over 40, in those with low educational attainment, and in those who were unemployed and/or had limited schooling. It’s interesting to note that the majority of cases under the age of 40 also had central obesity, and even people with underweight or normal BMI could develop central obesity and IFG. Promoting healthy lifestyle practices like consistent exercise and a balanced diet can help control and avoid central obesity and its associated dangers. This finding related with study conducted in Ethiopia that found the prevalence of central obesity among women was 49% based on waist circumference [22]. The incidence of central obesity in men and women grows with age, according to Finnish research, and it is linked to poor glucose tolerance [23]. Women tend to be more susceptible to central obesity and IFG due to complex molecular factors. Women tend to be more susceptible to central obesity and IFG due to complex molecular factors by a combination of hormonal, genetic, lifestyle, stress, and inflammatory factors. Women experience hormonal changes, such as the onset of menopause, which can lead to the development of central obesity and IFG [24]. Research has shown that non-overweight women who are vulnerable to the effects of stress are more likely to have excess abdominal fat and higher levels of the stress hormone cortisol [25]. Research also has shown that genetic factors play a significant role in the development of central obesity and IFG in women. Certain genetic variants are more common in women, which can increase their risk of developing this condition. Studies have identified several genes associated with central obesity and IFG, including ADAMTS9, TBX15-WARS2, and DNM3-PIGC [26].

These findings suggest that the complexity of hormonal and genetic interactions is the main cause of metabolic imbalances that influence the risk of IFG and central obesity in women. Central obesity, which is characterized by the accumulation of excess fat in the abdominal area, can cause insulin resistance, which is the main risk factor for IFG [27]. Estrogen, which is a female sex hormone, has a major influence on sugar and fat metabolism [28]. Estrogen can increase insulin sensitivity, which helps regulate blood sugar levels, as well as reduce fat accumulation in the midsection [29]. However, hormonal changes that occur during the menstrual cycle, pregnancy and menopause can cause fluctuations in insulin response, which can increase the risk of IFG and central obesity [30]. Insulin resistance is a common condition in women, and can occur throughout a woman’s life cycle from pregnancy, puberty, to menopause [31].

Research conducted in Finland also showed that the prevalence of central obesity increases with age in both men and women, and this is associated with abnormal glucose tolerance in the three BMI Timo categories [23]. Obesity, as measured by BMI, was found to increase the risk of IFG by 122% in Chinese adults after adjusting for other factors [32]. Central obesity has also been shown to have a stronger association with diabetes in the Chinese population compared with overall obesity [33]. There is an urgent need for interventions to reduce central obesity through the introduction of a weight-loss program, particularly for people with BMI within the normal range but significant central obesity.

This study reveals an association between central obesity, IFG, and a number of risk variables, such as levels of physical activity, aberrant lipid profiles, and hypertension. Gender, age, blood pressure, and HDL-C levels appear to be the key risk factors for central obesity and IFG. Numerous studies consistently show that central obesity, characterized by the accumulation of visceral fat, contributes to insulin resistance and the development of IFG [30–34]. This association is mediated through various biomolecular mechanisms, including the release of pro-inflammatory cytokines and adipokines from adipose tissue, which interfere with insulin signalling pathways [35]. Elevated blood pressure is also associated with insulin resistance and impaired glucose metabolism through mechanisms involving endothelial dysfunction and oxidative stress [36]. Dyslipidemia, characterized by high levels of total cholesterol, low-density lipoprotein cholesterol (LDL-C), and triglycerides, as well as low levels of high-density lipoprotein cholesterol (HDL-C), is a major risk factor for metabolic disorders such as IFG, because it disrupts lipid metabolism and insulin action at the molecular level [37].

At the molecular level, increased LDL-C triggers the accumulation of cholesterol in cells, disrupts insulin activity in regulating glucose transport into cells and activates inflammatory pathways [38]. High triglycerides can inhibit insulin sensitivity in target cells and damage endothelial function, while low HDL-C levels cannot transport excess cholesterol from cells, which can trigger oxidative stress and chronic inflammation [39]. In addition, dyslipidemia can trigger fat accumulation in liver and muscle cells, impairing glucose use and causing insulin resistance [40]. All these together create an adverse cellular environment, which disrupts glucose metabolism and causes IFG and increases the risk of developing type 2 diabetes. Therefore, understanding the mechanisms of dyslipidemia at the molecular and cellular level is essential in the treatment and prevention of metabolic disorders such as IFG.

This study looked into the dietary patterns and smoking behaviours, and it found significant differences between those with central obesity and concurrent abnormalities of IFG and those with noncentral obesity. The majority of those in the first group consumed these risky foods more than 6 times a week. They also consumed sweet foods, sweet drink, and soft drinks more frequently. In addition, key distinctions between noncentral and central obesity are made by the use of fatty foods, soft drinks, fruit, and smoking habits. Frequent use of fatty meals and soft drinks, which are renowned for having high quantities of harmful fats and added sugars, has long been linked to a higher risk of developing central obesity [41]. Instead, research highlights the benefits of a diet high in fruit, which provides vitamins, fibre, and natural sugars, enhancing satiety and metabolic health [42]. There is evidence to suggest that a diet high in added sugars promotes the development of obesity [43]. Sugar intake has been linked to an increased prevalence of childhood overweight/obesity [44]. Excessive consumption of unhealthy foods and sweetened soft drinks has been linked to weight gain, as they provide a large source of unnecessary calories. For more than 50 years, there has been evidence of increased consumption of sweet foods in overweight humans compared with those of normal weight [45]. It’s interesting to note that only smoking habit continued to be a significant risk factor in multivariate analysis for central obesity and IFG, emphasizing the need of addressing smoking habit in the context of metabolic health. Cigarettes contain toxic substances such as nicotine and tar which can damage cells, including cells that play a role in glucose regulation and lipid metabolism. Apart from that, smoking can also trigger chronic inflammation at the cellular level, which can interfere with the work of insulin cells [46].

Even though it provides a significant contribution, this research still has limitations that need to be considered. First, the cross-sectional research design limits its ability to establish causal association between variables, making it difficult to determine the direction of causality. Additionally, these studies rely on subjects’ memory and social desirability bias, thereby affecting the accuracy of reported behaviour. Small sample sizes for certain subgroups, such as underweight and centrally obese individuals and IFG, may also limit the precision of the findings. Although multivariate analyses adjust for potential confounding variables, there may still be unmeasured confounders that influence the reported associations. The ability to generalize the findings of this study is limited by a number of important factors and potential weaknesses. The 1,381 patients in the study sample offer in-depth information about the association between central obesity, impaired fasting glucose (IFG), and other risk variables. However, when considering how the results of this study can be applied to a larger population, we must be careful in interpreting them. The relative uniformity of the demographics of the study population is a significant weakness, as most participants with central obesity and IFG were female, over 40 years of age, and had certain marital, educational, and vocational characteristics. The results of this study may be influenced by variations in family history of diabetes mellitus, history of gestational diabetes mellitus, lifestyle, food practices, and health habits in certain regions. Therefore, cultural and geographical factors may also play a role. Future research should seek to collect more diverse and representative samples, account for cultural and regional diversity, and use longitudinal designs to analyse temporal patterns appropriately to maximize the generalizability of these findings. Despite these issues, this study offers insights that can guide future research and health care policy by laying a strong foundation for understanding the association between central adiposity, IFG, and related factors.

## Conclusion

The prevalence of central obesity with IFG is quite high, reaching 35.51% of the sample studied. There are significant differences in the characteristics of central obesity and IFG subjects, including gender, age, marital status, education level and occupation. Diet and smoking patterns also have a significant impact; individuals with central obesity and IFG are more likely to habitually consume high-risk foods and smoke. The results of multivariate analysis reveal that the most significant risk factors are gender (female), age over 40 years, high blood pressure, and low HDL-C levels.

## Supporting information

S1 Data (DTA)

S1 File (DOCX)
