# Supplementary material for: The characteristics and risk of obesity central and concomitant impaired fasting glucose: Findings from a cross-sectional study
Source: PLoS One. 2024 Jun 25;19(6):e0305604. doi: 10.1371/journal.pone.0305604 (PMC11198825; doi:10.1371/journal.pone.0305604)
Supplement: S1 File — (DOCX) [file pone.0305604.s002.docx]

STROBE Statement—checklist of items that should be included in reports of observational studies

|  | | Item No. | Recommendation | Page  No. | | Relevant text from manuscript | | |
| --- | --- | --- | --- | --- | --- | --- | --- | --- |
| **Title and abstract** | | 1 | (*a*) Indicate the study’s design with a commonly used term in the title or the abstract | 1  line 2 | | In title: “Findings from a cross-sectional study” | | |
|  |  |  | (*b*) Provide in the abstract an informative and balanced summary of what was done and what was found | 2  Line 48-65 | | In methods:  This study is based on a cross sectional of 1,381 population-based from Palembang, Indonesia.  Found/result:  The number of subjects consisting of 798 noncentral obesity with normoglycemia, 376 central obesity with normoglycemia, and 207 central obesity with concomitant IFG. The prevalence central obesity with concomitant IFG was 35.51%. In subjects with central obesity, there were significant differences in proportions based on sex, age, marital status, education, and occupation. In multivariate analysis show that the risk factors that contribute to having a significant association with central obesity with concomitant IFG are sex (female), age (>40 years), blood pressure (hypertension), and HDL-C <50 mg/dL (p<0.001). The analysis also founded that there was a significant difference in the dietary pattern of sweet foods (p=0.018), sweet drinks (p=0.002), soft drinks (p=0.001) and smoking habit (p<0.001) between subjects with obesity central and concomitant IFG compared to subjects with noncentral obesity. The majority of subjects with obesity central and concomitant IFG had consuming these risky foods >6 times/week. | | |
| Introduction | | | | | |  |  |  |
| Background/rationale | | 2 | Explain the scientific background and rationale for the investigation being reported | 3  Line 83-115 | | Obesity is an important risk factor for insulin resistance and hypertension which plays a central role in metabolic syndrome. The pathogenesis of diabetes and obesity is similar and related pathways of insulin resistance, oxidative stress (Ox-S), and pro-thrombotic and pro-inflammatory patterns [1]. The obesogenic environment stimulates overnutrition causing dysregulation of metabolic balance and ectopic fat accumulation in organs, such as the endothelium, liver, and skeletal muscle can lead to various metabolic disorders and diseases, including insulin resistance, glucose intolerance, diabetes, cardiovascular disease, and cerebrovascular disease [2]–[5].  Obesity is associated with concomitant chronic conditions, ranging from diabetes, dyslipidemia, to poor mental health. Its impact on the risk of stroke and cardiovascular disease, certain cancers, and osteoarthritis [6]–[9]. Some concomitants and risks are modifiable conditions, so lifestyle changes are needed to reduce or delay the progression of concomitants. At the same time, in many countries including Indonesia, economic growth and social changes have triggered shifts in dietary intake and physical activity at the population level, resulting in a significant increase in diabetes prevalence [10].  An early metabolic consequence of obesity is disruption of glucose and insulin homeostasis [11]. One of the consequences is impaired fasting glucose (IFG), which was introduced in the late 1990s by the American Diabetes Association (ADA) and the World Health Organization (WHO) as a stage of pre-diabetes referring to the level of fasting plasma glucose concentration above the upper normal range, but below the diagnostic limit of diabetes. The glucose range for IFG differs between organizations: the ADA glucose range is 5.6-6.9 mmol/L, while the WHO has more stringent criteria of 6.1-6.9 mmol/L. Both criteria are used and there is no consensus on IFG, especially not in children and adolescents. [12]. In adults, IFG is a predictor of type 2 diabetes [14], [15]. The mechanisms behind IFG are still not fully understood, but IFG results from impaired insulin secretion, which indicates beta cell dysfunction as well as increased hepatic glucose output [16]. Several studies reported the combination of general obesity and IFG was more strongly associated with diabetes risk than other studies with the same number of components [17], [18]. As visceral fat is metabolically more harmful than subcutaneous fat, but few information is available regarding the association between the risk of abnormal glucose in overweight and obese individuals and increased waist circumference [19]. Therefore, this study was conducted to identify the factors associated for obesity with concomitant IFG. Identifying the risk of IFG in obesity allows intervention with earlier precision. | | |
| Objectives | | 3 | State specific objectives, including any prespecified hypotheses | 3  Line 113-115 | | Therefore, this study was conducted to identify the factors associated for obesity with concomitant IFG. Identifying the risk of IFG in obesity allows intervention with earlier precision. | | |
| Methods | | | | | |  |  |  |
| Study design | | 4 | Present key elements of study design early in the paper | 4  Line 119-120 | | This cross-sectional study involved subjects aged >18 years from Palembang, Indonesia during July-December 2022. | | |
| Setting | | 5 | Describe the setting, locations, and relevant dates, including periods of recruitment, exposure, follow-up, and data collection | 4  Line 119-121 | | This cross-sectional study involved subjects aged >18 years from Palembang, Indonesia during July-December 2022. The multistage cluster random sampling method was used to select a representative sample of the population | | |
| Participants | | 6 | (*a*) *Cohort study*—Give the eligibility criteria, and the sources and methods of selection of participants. Describe methods of follow-up  *Case-control study*—Give the eligibility criteria, and the sources and methods of case ascertainment and control selection. Give the rationale for the choice of cases and controls  *Cross-sectional study*—Give the eligibility criteria, and the sources and methods of selection of participants | 4  Line 120-132 | | The multistage cluster random sampling method was used to select a representative sample of the population. From a total of 17 sub-districts, Seberang Ulu and Ilir were chosen at random as the first two sub-districts. Two of the five Seberang Ulu subdistricts and two of the twelve Seberang Ilir subdistricts were randomly chosen for the second stage and 25% of the villages from each subdistrict that was chosen were randomly chosen for the third stage. All households with household members older than 18 years old were identified in the final step. The eligibility requirements: be older than 18 and consent to taking fasting glucose and lipid profile tests as well as physical exams measuring their body weight, height, blood pressure, abdominal circumference, and waist circumference. The following were the exclusion criteria for cases and controls: 1) have fasting plasma glucose >126 mg/dl; 2) currently taking oral hypoglycemic medications; 3) taking any medication that could affect how glucose, insulin, or high-density lipoprotein cholesterol are metabolized; and 4) taking any medication for obesity. | | |
| Variables | | 7 | Clearly define all outcomes, exposures, predictors, potential confounders, and effect modifiers. Give diagnostic criteria, if applicable | 5  Line 150-165 | | Body mass index (BMI) was calculated by dividing body weight (kg) by body height (m^2^). By WHO criteria: Underweight (BMI < 18.50 kg/m^2^ ), normal weight (BMI = 18.50 to 24.99 kg/m^2^ ), overweight (BMI = 25.0 to 29.99 kg/m_2_ ), obese (BMI = >30.0). Waist circumference (WC) measurement was taken in the standing position, at the midpoint between the iliac crest and the least palpable rib precisely using non-stretchable tape. Central obesity was defined according to the WHO criteria: WC ≥ 94 cm for men and ≥ 80 cm for women.  Dietary pattern data was obtained from the quantitative food frequency questionnaire (FFQ). A photo album was used to help patients choose portion sizes. The reported intake was converted into daily consumption. Physical activity was measured using questionnaire from the International Physical Activity Questionnaire (IPAQ) [20]. The IPAQ assesses physical activity in four domains: leisure-time physical activity, household and yard activities, work-related physical activity, and transportation-related physical activity. Individuals who spent less than 600 metabolic equivalent minutes (METs) per week, which is the definition of low physical activity, were classified as inactive for this analysis. To simplify reporting, individuals who report more than 600 MET minutes per week will be referred to as “active”, whereas those with less than 600 MET minutes per week are “less active”. | | |
| Data sources/ measurement | | 8* | For each variable of interest, give sources of data and details of methods of assessment (measurement). Describe comparability of assessment methods if there is more than one group | 4  Line 134-147 | | The ethical guidelines established by the institutional research committee are followed in all procedures involving human beings. Through the use of questionnaires, physical exams, and blood testing, data were gathered through interviews. A standard questionnaire, information sheet, and consent form are distributed by the research team when they visit the participant's house. If participants concur, the examination was held the following day. A blood sample was taken during a fasting period of at least eight to twelve hours. Using standardized, laboratory techniques, the levels of plasma glucose, serum total, low-density lipoprotein and high-density lipoprotein cholesterol (LDL-c and HDL-c, respectively), and triglyceride (TG) were determined. Health professionals who have previously received training carry out physical examinations, taking blood pressure and anthropometric measurements after the wearer removes their bulky clothing, belts and shoes according to examination standards. Measures of blood pressure made with a sphygmomanometer, a common piece of medical equipment. Using a questionnaire, information about sex, age, education, occupation, marital status, and dietary pattern was gathered. | | |
| Bias | | 9 | Describe any efforts to address potential sources of bias | 4  Line 139-146 | | The effort for address potential information bias:  A standard questionnaire, information sheet, and consent form are distributed by the research team when they visit the participant's house. If participants concur, the examination was held the following day. A blood sample was taken during a fasting period of at least eight to twelve hours. Using standardized, laboratory techniques, the levels of plasma glucose, serum total, low-density lipoprotein and high-density lipoprotein cholesterol (LDL-c and HDL-c, respectively), and triglyceride (TG) were determined. Health professionals who have previously received training carry out physical examinations, taking blood pressure and anthropometric measurements after the wearer removes their bulky clothing, belts and shoes according to examination standards. Measures of blood pressure made with a sphygmomanometer, a common piece of medical equipment. | | |
| Study size | | 10 | Explain how the study size was arrived at | N/A | | Not state in article:  The study size was calculated based on the reference estimate for prediabetes in Palembang City from a previous study, 27.8% [2], maximum error 5%.  $n=\frac{{(Z_{1-\frac{\alpha}{2}})}^{2} p(1-p)}{\left( d \right)^{2}}$  $n=\frac{{1,96}^{2}(0,278)(1-0,278)}{\left( 0,05 \right)^{2}}$ = 308,4 for each group  Where:  n = minimum number of samples required  Z_1-α/2_ = significance level of 5% = 1.96  p = prevalence of prediabetes estimate from previous research was 27.8%  d = tolerable absolute error | | |
| Quantitative variables | | 11 | Explain how quantitative variables were handled in the analyses. If applicable, describe which groupings were chosen and why | 4  Line 167-173 | | **Statistical Analysis**  The statistical analysis was performed using the statistical program STATA version 15 (College Station, Texas 77845 USA). Categorical data represented as counts of frequencies with n (%). Multinomial logistic regression to analyze factors that were associated outcome variable (i.e., the present obesity central and IFG status) consisted of three categories: 0 = noncentral obesity normoglycemic and, 1 = central obesity with normoglycemic, 2= central obesity with IFG. | | |
| Statistical methods | | 12 | (a) Describe all statistical methods, including those used to control for confounding | 4  Line 173-178 | | An adjusted multinomial logistic regression models were used to identify the potential factors that have a significant role in the higher risk of obesity and concomitant IFG. We checked the multicollinearity among the explanatory variables using variance inflation factor (VIF). VIF value ≤ 2.0 indicates absence of multicollinearity [21]. The data was summarized with relative risk ratios (RRR) and 95 % confidence interval. Results were considered significant when p < 0.05. | | |
|  | |  | (b) Describe any methods used to examine subgroups and interactions | N/A | |  | | |
|  | |  | (c) Explain how missing data were addressed | N/A | |  | | |
|  | |  | (d) Cross-sectional study—If applicable, describe analytical methods taking account of sampling strategy | N/A | |  | | |
|  | |  | (e) Describe any sensitivity analyses | N/A | |  | | |
| Results | | | | | | | |  |
| Participants | | 13* | (a) Report numbers of individuals at each stage of study—eg numbers potentially eligible, examined for eligibility, confirmed eligible, included in the study, completing follow-up, and analysed | 6  Line 187-191 | | The total number of subjects in this study was 1,381 consisting of 798 noncentral obesity with normoglycemia, 376 central obesity with normoglycemia, and 207 central obesity with concomitant IFG. Table 1 provides the characteristics of subjects with obesity and concomitant IFG. Based on the table, the prevalence central obesity with concomitant IFG was 35.51% (207 out of 583). | |  |
|  |  |  | (b) Give reasons for non-participation at each stage | N/A | |  | |  |
|  |  |  | (c) Consider use of a flow diagram | N/A | |  | |  |
| Descriptive data | | 14* | (a) Give characteristics of study participants (eg demographic, clinical, social) and information on exposures and potential confounders | 6  Line 189-197 | | Table 1 provides the characteristics of subjects with obesity and concomitant IFG. Based on the table, the prevalence central obesity with concomitant IFG was 35.51% (207 out of 583). Data analysis in this study took subjects with noncentral obesity with normoglycemia as a reference group for comparison. In subjects with central obesity, there were significant differences in proportions based on sex, age, marital status, education, and occupation. Subjects with central obesity with concomitant IFG were predominantly female (20.39%), aged >=40 years (20%), with marital status of death divorce (32%), no school (18.29%), and not working (20.96). It is interesting that central obesity was possessed by 25.63% of subjects <40 years of age and another 9.12% with central obesity and IFG. | |  |
|  |  |  | (b) Indicate number of participants with missing data for each variable of interest | N/A | |  | |  |
|  |  |  | (c) *Cohort study*—Summarise follow-up time (eg, average and total amount) |  | |  | |  |
| Outcome data | | 15* | *Cohort study*—Report numbers of outcome events or summary measures over time |  | |  | |  |
|  |  |  | *Case-control study—*Report numbers in each exposure category, or summary measures of exposure |  | |  | |  |
|  |  |  | *Cross-sectional study—*Report numbers of outcome events or summary measures | 6  Line 187-189 | | The total number of subjects in this study was 1,381 consisting of 798 noncentral obesity with normoglycemia, 376 central obesity with normoglycemia, and 207 central obesity with concomitant IFG. | |  |
| Main results | | 16 | (*a*) Give unadjusted estimates and, if applicable, confounder-adjusted estimates and their precision (eg, 95% confidence interval). Make clear which confounders were adjusted for and why they were included | 6  Line 209-214  7  Line 215-225 | | Although partially or bivariate, physical activity, total cholesterol >200 mg/dL, LDL-C >160 mg/dL, triglyceride >150 mg/dL had a significant association with central obesity with concomitant IFG (p<0.001). In multivariate analysis (table 2) show that the risk factors that contribute to having a significant association with central obesity with concomitant IFG are sex (female), age (>40 years), blood pressure (hypertension), and HDL-C <50 mg/dL (p<0.001).  This study also explored the dietary pattern of the subjects (table 3), the results of the analysis showed that there was a significant difference in the consumption frequency pattern of sweet foods (p=0.018), sweet drinks (p=0.002), soft drinks (p=0.001) and smoking habit (p<0.001) between subjects with obesity central and concomitant IFG compared to subjects with noncentral obesity. The majority of subjects with obesity central and concomitant IFG had the habit of consuming these risky foods >6 times/week. Fatty food (p=0.005), soft drink (p=0.001), fruit consumption (p=0.008), and smoking habit (p<0.001) were significant variables that distinguished noncentral obesity and central obesity subjects. Interestingly (table 4), although bivariate multiple food consumption was a risk for central obesity with concomitant IFG, on multivariate only smoking habit was significant as a risk with both non-daily (p=0.004) and daily smokers (<0.001) when compared to subjects without central obesity. | |  |
|  |  |  | (*b*) Report category boundaries when continuous variables were categorized | N/A | | . | |  |
|  |  |  | © If relevant, consider translating estimates of relative risk into absolute risk for a meaningful time period | N/A | |  | |  |
|  |  |  | Report other analyses done—eg analyses of subgroups and interactions, and sensitivity analyses | N/A | |  | |  |
| Discussion | | | | | | | |  |
| Key results | 18 | Summarise key results with reference to study objectives | | 7  Line 228-232 | In 35.51% of the sample (table 1), central obesity and impaired fasting glucose (IFG) were present. This condition was more common in women over 40, in those with low educational attainment, and in those who were unemployed and/or had limited schooling. It's interesting to note that the majority of cases under the age of 40 also had central obesity, and even people with underweight or normal BMI could develop central obesity and IFG. | | |  |
| Limitations | 19 | Discuss limitations of the study, taking into account sources of potential bias or imprecision. Discuss both direction and magnitude of any potential bias | | 9  Line 313-320 | Even though it provides a significant contribution, this research still has limitations that need to be considered. First, the cross-sectional research design limits its ability to establish causal association between variables, making it difficult to determine the direction of causality. Additionally, these studies rely on subjects' memory and social desirability bias, thereby affecting the accuracy of reported behaviour. Small sample sizes for certain subgroups, such as underweight and centrally obese individuals and IFG, may also limit the precision of the findings. Although multivariate analyses adjust for potential confounding variables, there may still be unmeasured confounders that influence the reported associations. | | |  |
| Interpretation | 20 | Give a cautious overall interpretation of results considering objectives, limitations, multiplicity of analyses, results from similar studies, and other relevant evidence | | 9-10  268-312 | This study reveals an association between central obesity, IFG, and a number of risk variables, such as levels of physical activity, aberrant lipid profiles, and hypertension. Gender, age, blood pressure, and HDL-C levels appear to be the key risk factors for central obesity and IFG. Numerous studies consistently show that central obesity, characterized by the accumulation of visceral fat, contributes to insulin resistance and the development of IFG [30]-[34]This association is mediated through various biomolecular mechanisms, including the release of pro-inflammatory cytokines and adipokines from adipose tissue, which interfere with insulin signalling pathways [35]. Elevated blood pressure is also associated with insulin resistance and impaired glucose metabolism through mechanisms involving endothelial dysfunction and oxidative stress [36]. Dyslipidemia, characterized by high levels of total cholesterol, low-density lipoprotein cholesterol (LDL-C), and triglycerides, as well as low levels of high-density lipoprotein cholesterol (HDL-C), is a major risk factor for metabolic disorders such as IFG, because it disrupts lipid metabolism and insulin action at the molecular level [37].  At the molecular level, increased LDL-C triggers the accumulation of cholesterol in cells, disrupts insulin activity in regulating glucose transport into cells and activates inflammatory pathways [38]. High triglycerides can inhibit insulin sensitivity in target cells and damage endothelial function, while low HDL-C levels cannot transport excess cholesterol from cells, which can trigger oxidative stress and chronic inflammation [39]. In addition, dyslipidemia can trigger fat accumulation in liver and muscle cells, impairing glucose use and causing insulin resistance [40]. All these together create an adverse cellular environment, which disrupts glucose metabolism and causes IFG and increases the risk of developing type 2 diabetes. Therefore, understanding the mechanisms of dyslipidemia at the molecular and cellular level is essential in the treatment and prevention of metabolic disorders such as IFG.  This study looked into the dietary patterns and smoking behaviours, and it found significant differences between those with central obesity and concurrent abnormalities of IFG and those with noncentral obesity. The majority of those in the first group consumed these risky foods more than 6 times a week. They also consumed sweet foods, sweet drink, and soft drinks more frequently. In addition, key distinctions between noncentral and central obesity are made by the use of fatty foods, soft drinks, fruit, and smoking habits. Frequent use of fatty meals and soft drinks, which are renowned for having high quantities of harmful fats and added sugars, has long been linked to a higher risk of developing central obesity [41]. Instead, research highlights the benefits of a diet high in fruit, which provides vitamins, fibre, and natural sugars, enhancing satiety and metabolic health [42]. There is evidence to suggest that a diet high in added sugars promotes the development of obesity [43]. Sugar intake has been linked to an increased prevalence of childhood overweight/obesity [44]. Excessive consumption of unhealthy foods and sweetened soft drinks has been linked to weight gain, as they provide a large source of unnecessary calories. For more than 50 years, there has been evidence of increased consumption of sweet foods in overweight humans compared with those of normal weight [45]. It's interesting to note that only smoking habit continued to be a significant risk factor in multivariate analysis for central obesity and IFG, emphasizing the need of addressing smoking habit in the context of metabolic health. Cigarettes contain toxic substances such as nicotine and tar which can damage cells, including cells that play a role in glucose regulation and lipid metabolism. Apart from that, smoking can also trigger chronic inflammation at the cellular level, which can interfere with the work of insulin cells [46]. | | |  |
| Generalisability | 21 | Discuss the generalisability (external validity) of the study results | | 9-10  320-336 | The ability to generalize the findings of this study is limited by a number of important factors and potential weaknesses. The 1,381 patients in the study sample offer in-depth information about the association between central obesity, impaired fasting glucose (IFG), and other risk variables. However, when considering how the results of this study can be applied to a larger population, we must be careful in interpreting them. The relative uniformity of the demographics of the study population is a significant weakness, as most participants with central obesity and IFG were female, over 40 years of age, and had certain marital, educational, and vocational characteristics. The results of this study may be influenced by variations in family history of diabetes mellitus, history of gestational diabetes mellitus, lifestyle, food practices, and health habits in certain regions. Therefore, cultural and geographical factors may also play a role. Future research should seek to collect more diverse and representative samples, account for cultural and regional diversity, and use longitudinal designs to analyse temporal patterns appropriately to maximize the generalizability of these findings. Despite these issues, this study offers insights that can guide future research and health care policy by laying a strong foundation for understanding the association between central adiposity, IFG, and related factors. | | |  |
| Other information | |  | | | | | |  |
| Funding | 22 | Give the source of funding and the role of the funders for the present study and, if applicable, for the original study on which the present article is based | | 10  Line 338-341 | The research of this article was funded by DIPA of Public Service Agency of Universitas Sriwijaya 2023. Number SP DIPA-023.17.2.677515/2023, on November 2022. In accordance with the Rector’s Decree Number:0188/UN9.3.1/SK/2023, On April 18, 2023. | | |  |

*Give information separately for cases and controls in case-control studies and, if applicable, for exposed and unexposed groups in cohort and cross-sectional studies.

**Note:** An Explanation and Elaboration article discusses each checklist item and gives methodological background and published examples of transparent reporting. The STROBE checklist is best used in conjunction with this article (freely available on the Web sites of PLoS Medicine at http://www.plosmedicine.org/, Annals of Internal Medicine at http://www.annals.org/, and Epidemiology at http://www.epidem.com/). Information on the STROBE Initiative is available at www.strobe-statement.org.
